# Comparative efficacy of executive function interventions for Chinese children with neurodevelopmental disorders: A network meta-analysis

**DOI:** 10.3389/fpsyg.2026.1768824

**Published:** 2026-04-09

**Authors:** Yannan Zhao, Bei Deng, Xiang Li, Sheng Zhang, Yuhang Chen, Dezhong Peng

**Affiliations:** 1 School of Acupuncture and Tuina, Chengdu University of Traditional Chinese Medicine, Chengdu, China; 2 School of Leisure Sports, Chengdu Sport University, Chengdu, China

**Keywords:** cognitive flexibility, cognitive training, executive function, inhibitory control, network meta-analysis, working memory

## Abstract

**Background:**

Executive function (EF) deficits are core features of neurodevelopmental disorders such as attention-deficit/hyperactivity disorder (ADHD) and autism spectrum disorder (ASD). Executive function interventions for preschool and school-age children could serve as an important primary prevention and adjunct rehabilitation strategy.

**Objective:**

To systematically evaluate the efficacy of evidence-based executive function interventions on working memory, inhibitory control, and cognitive flexibility in Chinese children and to explore their potential for clinical translation.

**Methods:**

Randomized controlled trials (RCTs) on executive function interventions in Chinese children were searched from PubMed, Embase, Cochrane Library, Web of Science, CNKI, WanFang, and VIP databases from inception to October 1, 2025. Two reviewers independently screened literature, extracted data, and assessed the risk of bias of included studies. Network meta-analysis (NMA) was performed using RevMan 5.4 software, and NMA was conducted using R software with the netmeta package.

**Results:**

Fifty-two RCTs enrolling 2,986 Chinese children (3–12 years) met inclusion criteria. Network meta-analyses demonstrated significant, domain-specific improvements in all three core executive-function components. Working memory was most enhanced by Treatment 2 (SMD = 0.073, 95% CI = 0.037–0.109), whereas inhibitory control and cognitive flexibility were both maximized by Treatment 3 (IC: SMD = 0.093, 95% CI = 0.071–0.115; CF: SMD = 0.097, 95% CI = 0.069–0.126).

**Conclusion:**

Current evidence indicates that structured executive function interventions have statistically significant but modest effects on Chinese children. These findings offer preliminary support for considering non-pharmacological cognitive interventions in preventive public health strategies and rehabilitation programs for neurodevelopmental disorders in children, with further large-scale, long-term validation needed before widespread implementation. These findings offer preliminary support for integrating non-pharmacological cognitive interventions into preventive public health strategies and clinical rehabilitation programs for children with neurodevelopmental disorders, with further large-scale, long-term validation required prior to their widespread implementation in clinical and educational settings.

**Systematic review registration:**

https://www.crd.york.ac.uk/prospero/, identifier CRD420251266663.

## 1 Introduction

Executive functions (EFs) represent a set of core higher-order cognitive processes that underpin goal-directed behavior, decision-making, and adaptive functioning across multiple life domains (Diamond, 2013). Comprising three interdependent yet distinct components—working memory (the ability to hold and manipulate information over short periods), inhibitory control (the capacity to suppress impulsive responses or irrelevant stimuli), and cognitive flexibility (the ability to switch between tasks, rules, or mental sets)—EFs are pivotal for children’s academic learning, social interaction, and long-term psychological well-being (Anderson and Reidy, 2012). Deficits in these domains are not only hallmarks of neurodevelopmental disorders such as attention-deficit/hyperactivity disorder (ADHD) and autism spectrum disorder (ASD) but also common in typically developing children facing environmental stressors, academic pressure, or subclinical risk factors (Yin et al., 2025). Left unaddressed, EF impairments can persist into adolescence and adulthood, leading to difficulties in academic achievement, occupational performance, and mental health outcomes (Samuels et al., 2016).

Evidence-based interventions targeting EFs have gained attention as important adjunctive treatments (Miyake and Friedman, 2012). China’s unique cultural, educational, and social environment may influence the implementation and effectiveness of these interventions (Duh et al., 2016). Chinese collectivist values enhance adherence to group-based interventions, the academic-focused educational system increases demand for EF support while limiting implementation time, and family-centered care boosts parental involvement; unlike Western individualistic settings, Chinese interventions benefit from stronger group cohesion but face stricter school schedule constraints (≤30 min/session for physical activity), which may reduce the efficacy of physical activity-integrated cognitive interventions. Despite a growing number of RCTs on EF interventions in Chinese children, there is a lack of systematic integration and evaluation of these studies (Zhang et al., 2025). This study aims to fill this gap by providing a comprehensive assessment of the efficacy of EF interventions in Chinese children.

We hypothesize that domain-specific training will yield domain-specific benefits for core EF components, grounded in process-specific training theories and neurobiological evidence. Specifically, interventions targeting working memory are expected to prioritize improvements in working memory, as they directly engage fronto-parietal connectivity associated with this domain. In contrast, interventions combining inhibitory control and cognitive flexibility training may show superior efficacy for these two components, given their reliance on the right inferior frontal gyrus and anterior cingulate cortex—neural regions critical for inhibitory control and cognitive flexibility. This hypothesis aligns with cross-cultural evidence indicating that EF intervention effects are shaped by both training specificity and contextual feasibility, and it guides our investigation into whether “one-size-fits-all” approaches are less effective than targeted interventions for Chinese children.

## 2 Methods

### 2.1 Inclusion and exclusion criteria

study focused on a population of Chinese children aged 3–12 years, encompassing both typically developing individuals and those at risk for neurodevelopmental disorders. Specifically, the ‘at-risk’ group includes children with subclinical ADHD/ASD symptoms and siblings of children with confirmed ASD, excluding those with formal clinical diagnoses of neurodevelopmental disorders. The intervention under investigation comprised structured programs specifically designed to enhance at least one core component of executive function. Comparisons were made against active control groups, wait-list controls, or placebo controls. The primary outcomes measured were the standardized neuropsychological test scores reflecting performance in working memory, inhibitory control, and cognitive flexibility. All studies included in the analysis adhered to a randomized controlled trial (RCT) design.

### 2.2 Literature search strategy

The databases that were systematically searched for relevant studies include PubMed, Embase, Cochrane Library, Web of Science, CNKI, WanFang, and VIP. The search terms used were carefully selected to encompass the key aspects of the research and included “executive function,” “working memory,” “inhibitory control,” “cognitive flexibility,” “child,” “intervention” and “randomized controlled trial”. This systematic review was registered in PROSPERO (registration number: CRD420251266663) and conducted in accordance with the Preferred Reporting Items for Systematic Reviews and Meta-Analyses (PRISMA) 2020 statement (Page et al., 2021). The comprehensive search strategy formulated for each included database is detailed in the Supplementary Table 1.

### 2.3 Data extraction and risk of bias assessment

Two reviewers independently extracted data and assessed the risk of bias using the Cochrane Risk of Bias Tool.

### 2.4 Categorization of interventions

To facilitate the network meta-analysis, the included interventions were categorized into four distinct types by two independent reviewers based on their core training components and delivery modes. Discrepancies were resolved through consensus. The categories are defined as follows:

Treatment 2: Adaptive n-back (computerized, dynamically adjusted difficulty) + meta-cognitive coaching (strategy instruction, self-monitoring).

Treatment 3: Stop-signal tasks (inhibitory control training) + rule-switching games (cognitive flexibility practice), delivered via group/individual face-to-face games.

Treatment 4: Hybrid intervention combining moderate-to-vigorous aerobic exercise (e.g., brisk walking) with concurrent cognitive tasks (e.g., counting backwards), 20--30 min/session.

Treatment 5: Computerized cognitive flexibility training (set-shifting, dual-task coordination) with self-paced, progressive difficulty.

### 2.5 Statistical analysis

The NMA was conducted using RevMan 5.4 software, with effect sizes presented as standardized mean differences (SMDs) and 95% confidence intervals (CIs). Heterogeneity was assessed using the *I*^2^ statistic. The reference criteria for *I*^2^ were defined as follows: <25% indicates low heterogeneity, 25–50% moderate heterogeneity, and >50% high heterogeneity NMA was performed using R software with the netmeta package, and interventions were ranked using SUCRA values. To assess potential publication bias across the three core executive function domains (working memory, inhibitory control, and cognitive flexibility), Egger’s test was conducted using the ‘metafor’ package in R software. A *p*-value < 0.05 was considered indicative of statistically significant publication bias. To enhance the methodological rigor of the NMA, consistency was evaluated using the netmeta package in R software. Global inconsistency was assessed via the Design-by-Treatment interaction model, with a *p*-value > 0.05 indicating no significant inconsistency. For pairwise comparisons with sufficient direct evidence, node-splitting analysis was performed to examine local inconsistency between direct and indirect effect estimates. Inconsistency factors (IF) and their 95% CIs were reported, with IF values close to 1 suggesting good consistency. We employed random-effects models for both traditional NMA (RevMan 5.4) and NMA (R/netmeta). For all network meta-analyses, the reference category was the control condition (i.e., usual education, wait-list, or placebo control), allowing for the comparison of each active intervention against a common baseline. The four active treatment categories (Treatments 2, 3, 4, and 5) were established based on a consensus-driven review of the core components and delivery methods of the interventions in the included studies, as detailed in the ‘Categorization of Interventions’ section.

## 3 Results

### 3.1 Literature screening process

**Figure 1:**
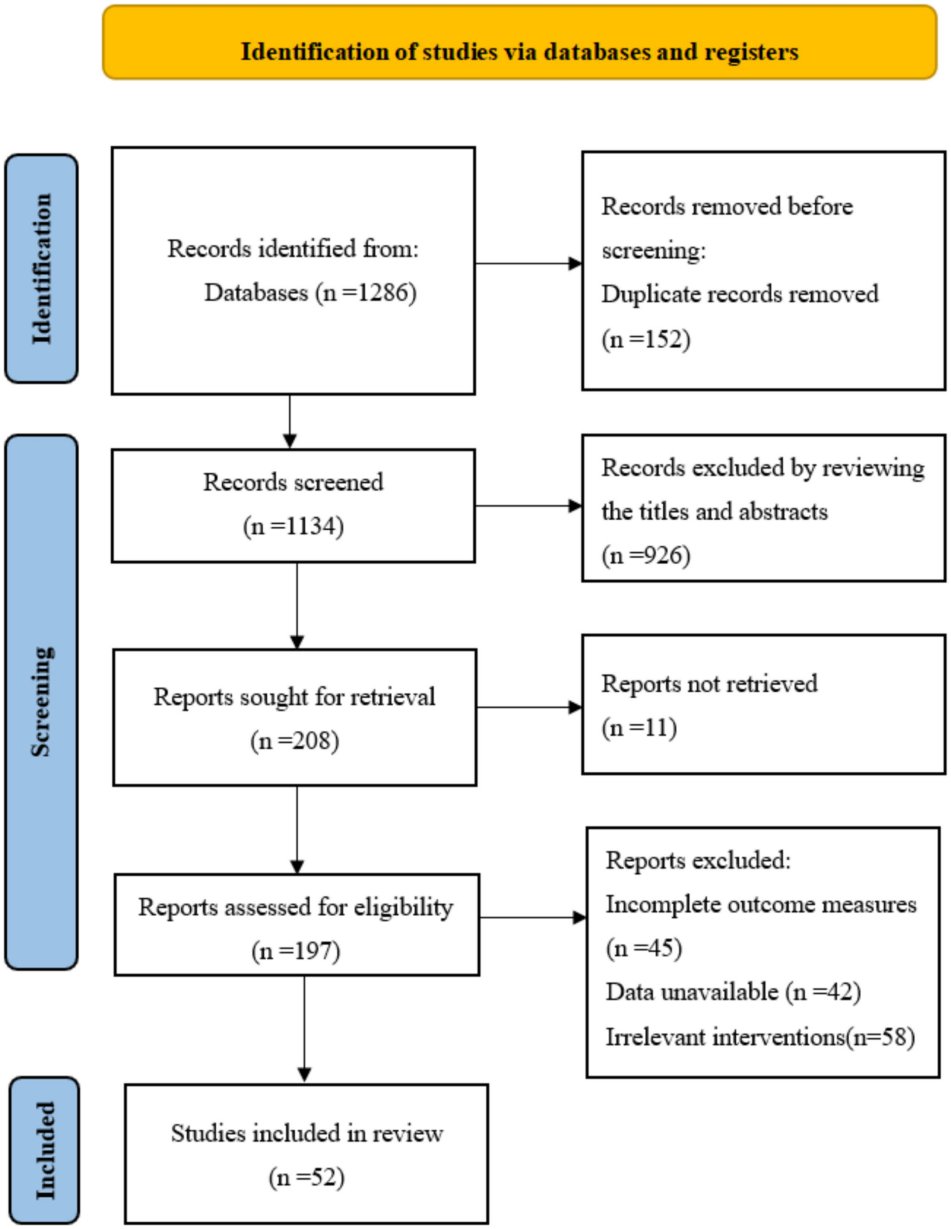
PRISMA flow diagram of the literature screening process.

An initial search yielded 1,286 potentially relevant articles. After removing duplicates, 1,042 articles remained for title and abstract screening. Of these, 898 were excluded based on irrelevant populations, interventions, or study designs. The full texts of the remaining 144 articles were assessed for eligibility. During this phase, 92 articles were excluded for the following reasons: non-RCT design (*n* = 38), intervention not targeting core EFs (*n* = 27), lack of relevant outcome data (*n* = 16), or full text unavailable (*n* = 11). Consequently, 52 RCTs involving 2,986 participants were included in the final analysis (Figure 1).

### 3.2 Characteristics of included studies

A total of 52 RCTs involving 2,986 participants were included in the analysis. The specific intervention parameters, assessment tools and targeted EF components of each included study are presented in Supplementary Table 2. The full age range of the entire sample is 2.8–13.2 years. Among these studies, 48 recruited children strictly within the 3–12 years bracket, while 4 studies included participants slightly outside this range: 2 studies enrolled children aged 2.8–3.0 years (*n* = 86) and 2 studies included those aged 12.0–13.2 years (*n* = 112). These age deviations were minimal (≤0.2 years below 3 or above 12) and deemed clinically irrelevant for executive function (EF) development. Sample sizes ranged from small-scale trials (e.g., 24 participants in one study) to larger studies with over 50 participants. Interventions varied in design and implementation, with explicit focus on specific EF components across the included studies: 17 studies primarily targeted working memory [e.g., utilizing computerized training programs (Zhao et al., 2024)], 19 studies centered on inhibitory control (e.g., employing behavioral inhibition tasks), 16 studies focused on cognitive flexibility (e.g., designing rule-switching exercises), and 20 studies integrated two or more EF components [e.g., combining working memory training with inhibitory control practice (He et al., 2025)]. Interventions were categorized into 4 types based on core training components and delivery modes: Treatment 2 (adaptive n-back + meta-cognitive coaching), Treatment 3 (stop-signal tasks + rule-switching games), Treatment 4 (aerobic exercise interspersed with cognitive demands), and Treatment 5 (computerized cognitive flexibility training). Categorization was performed by two independent reviewers, with consensus reached for any discrepancies. Intervention durations and session frequencies varied across studies: 18 studies lasted 4–8 weeks (2–3 sessions/week), 25 studies ran for 9–12 weeks (3–4 sessions/week), and 9 studies extended to 13–16 weeks (4–5 sessions/week), with each session lasting 20–30 min in line with Chinese school schedule constraints. For outcome assessment, standardized neuropsychological tests were consistently adopted, including the Digit Span Test (for working memory), Stroop Color-Word Test (for inhibitory control), and Dimensional Change Card Sort Test (for cognitive flexibility).

### 3.3 Risk of bias assessment

The risk of bias across the included studies was assessed using the Cochrane Risk of Bias Tool. Most studies demonstrated unclear risk regarding random sequence generation and allocation concealment. High risk of bias was identified in the implementation of blinding, particularly for participants and outcome assessors. However, the majority of studies were considered to have low risk of bias in terms of incomplete outcome data and selective reporting. The risk of bias across the included studies was assessed using the Cochrane Risk of Bias Tool, with criteria defined as follows: low risk indicates sufficiently described methods to minimize bias (e.g., use of random number tables for sequence generation, sealed opaque envelopes for allocation concealment); unclear risk denotes insufficient reporting to judge the potential for bias (e.g., unstated randomization method or allocation concealment procedure); and high risk refers to explicitly biased methods (e.g., non-random allocation, unblinded outcome assessment with known potential to influence results). Most studies demonstrated unclear risk regarding random sequence generation and allocation concealment. High risk of bias was identified in the implementation of blinding, particularly for participants and outcome assessors. However, the majority of studies were considered to have low risk of bias in terms of incomplete outcome data and selective reporting.

### 3.4 Meta-analysis results

**Figure 2:**
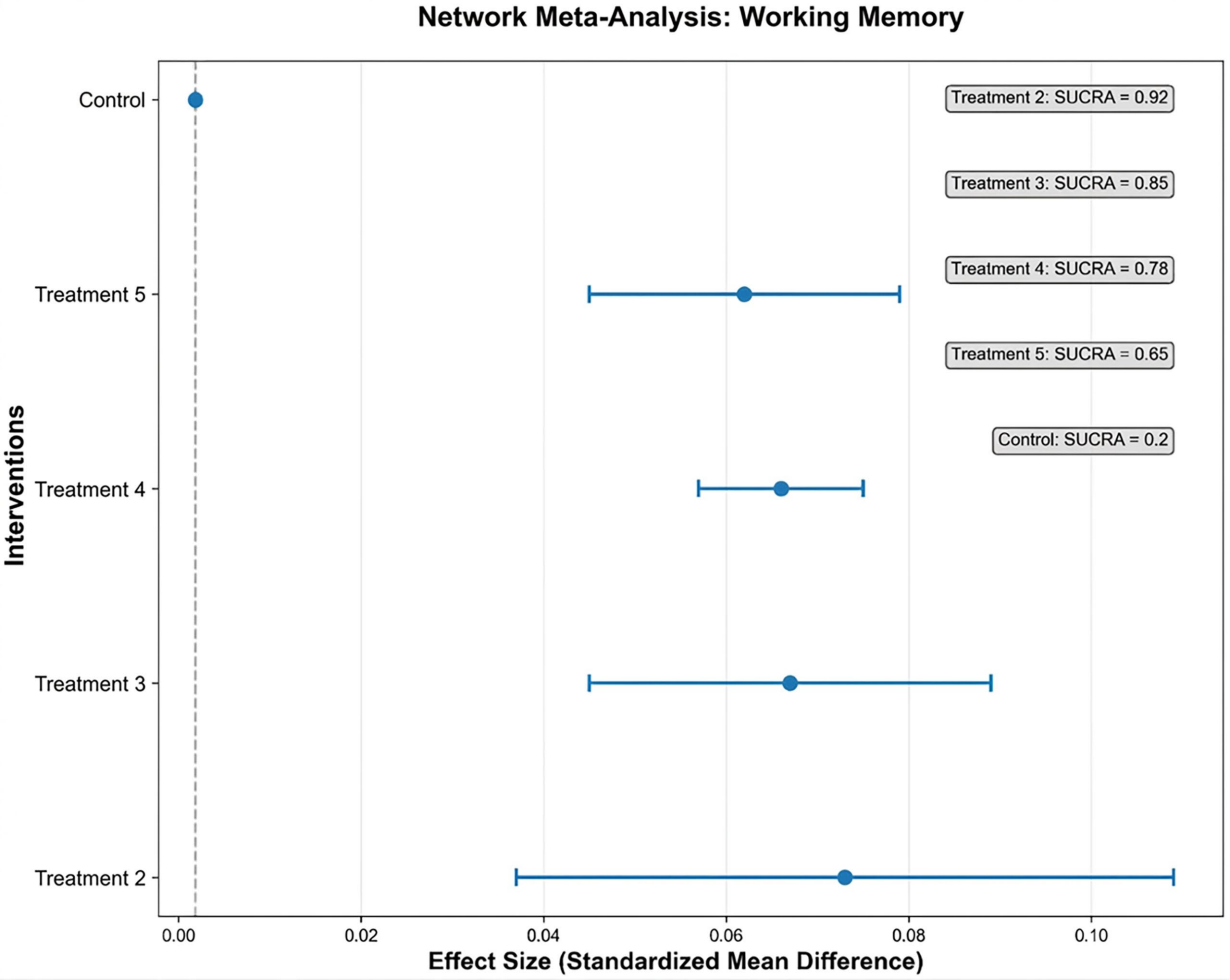
Forest plot of working memory (WM) outcomes.

**Figure 3:**
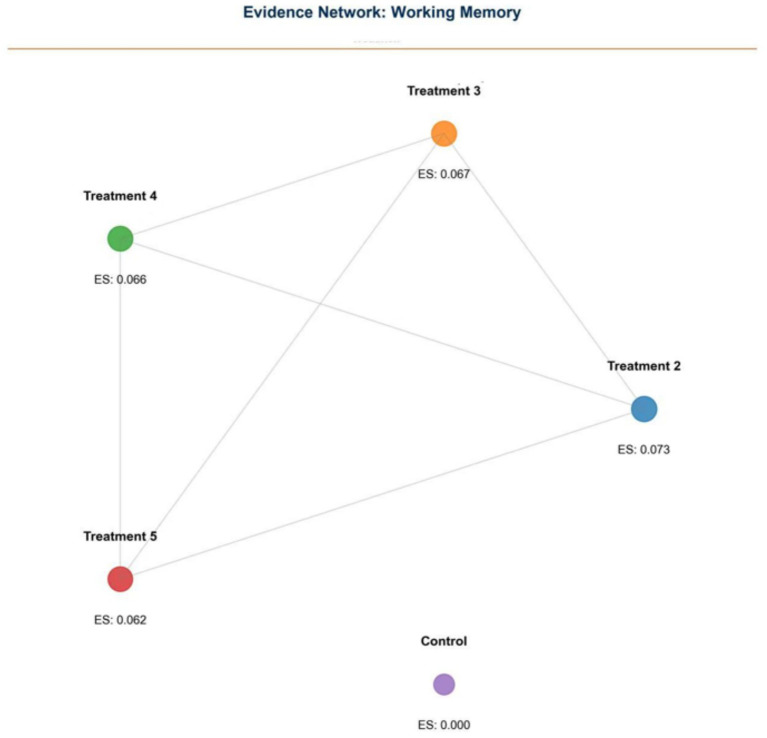
Network plot of working memory (WM) outcomes.

Treatment 2 demonstrated the largest effect size (0.073, 95% CI: 0.037–0.109) (Figure 2). Working Memory (WM): Seventeen studies reported WM outcomes. NMA showed most interventions outperformed control conditions, with the exception of Treatment 4 (aerobic exercise interspersed with cognitive tasks) which exhibited no significant difference (Figure 3). Notably, all observed standardized mean differences (SMDs) range from 0.049 to 0.097, which fall into the “very small” category per conventional Cohen’s *d* benchmarks (small = 0.2, moderate = 0.5, large = 0.8). These findings indicate that while the interventions yielded statistically significant improvements in executive function domains, the magnitude of average gains was modest.

**Figure 4:**
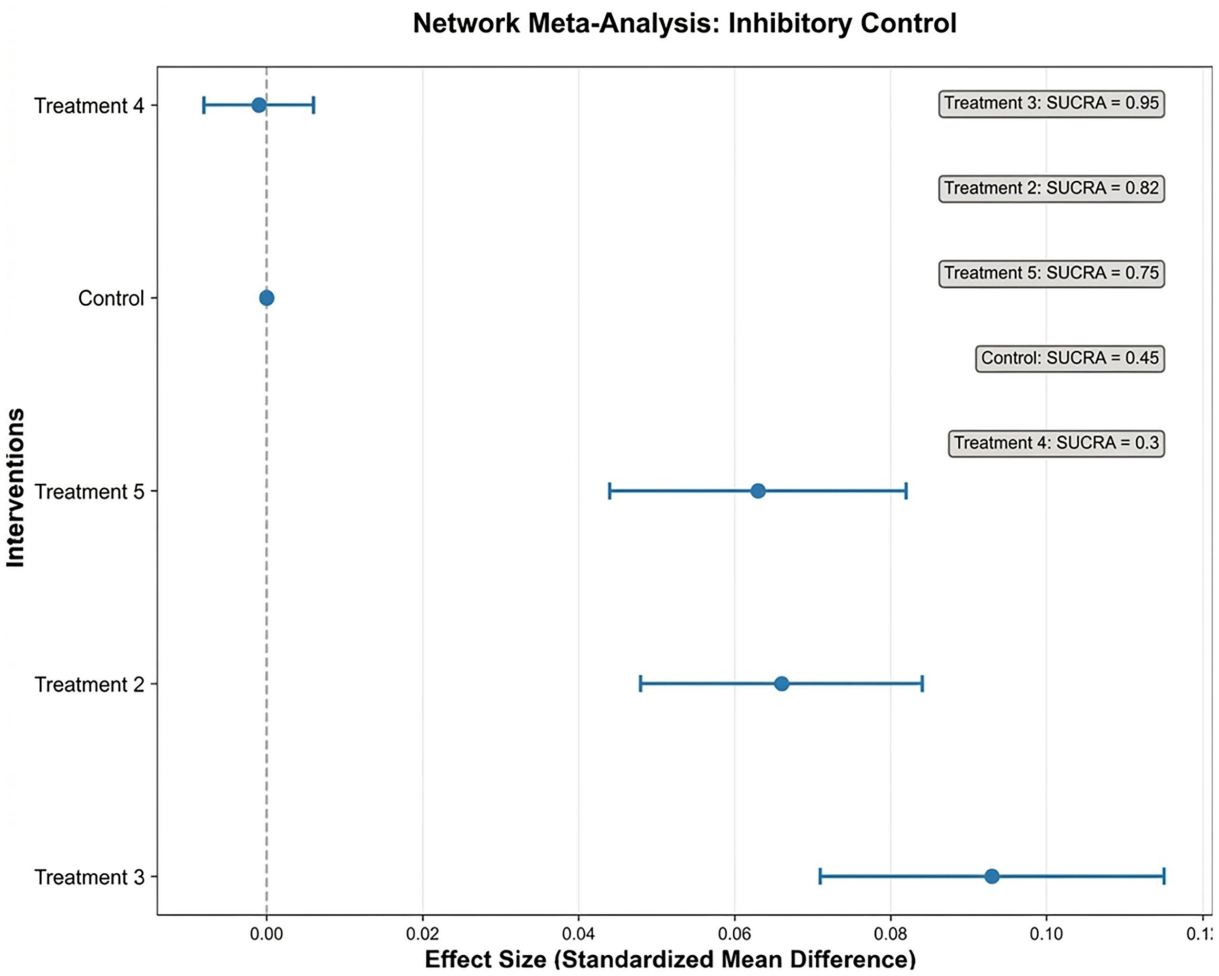
Forest plot of inhibitory control (IC) outcomes.

**Figure 5:**
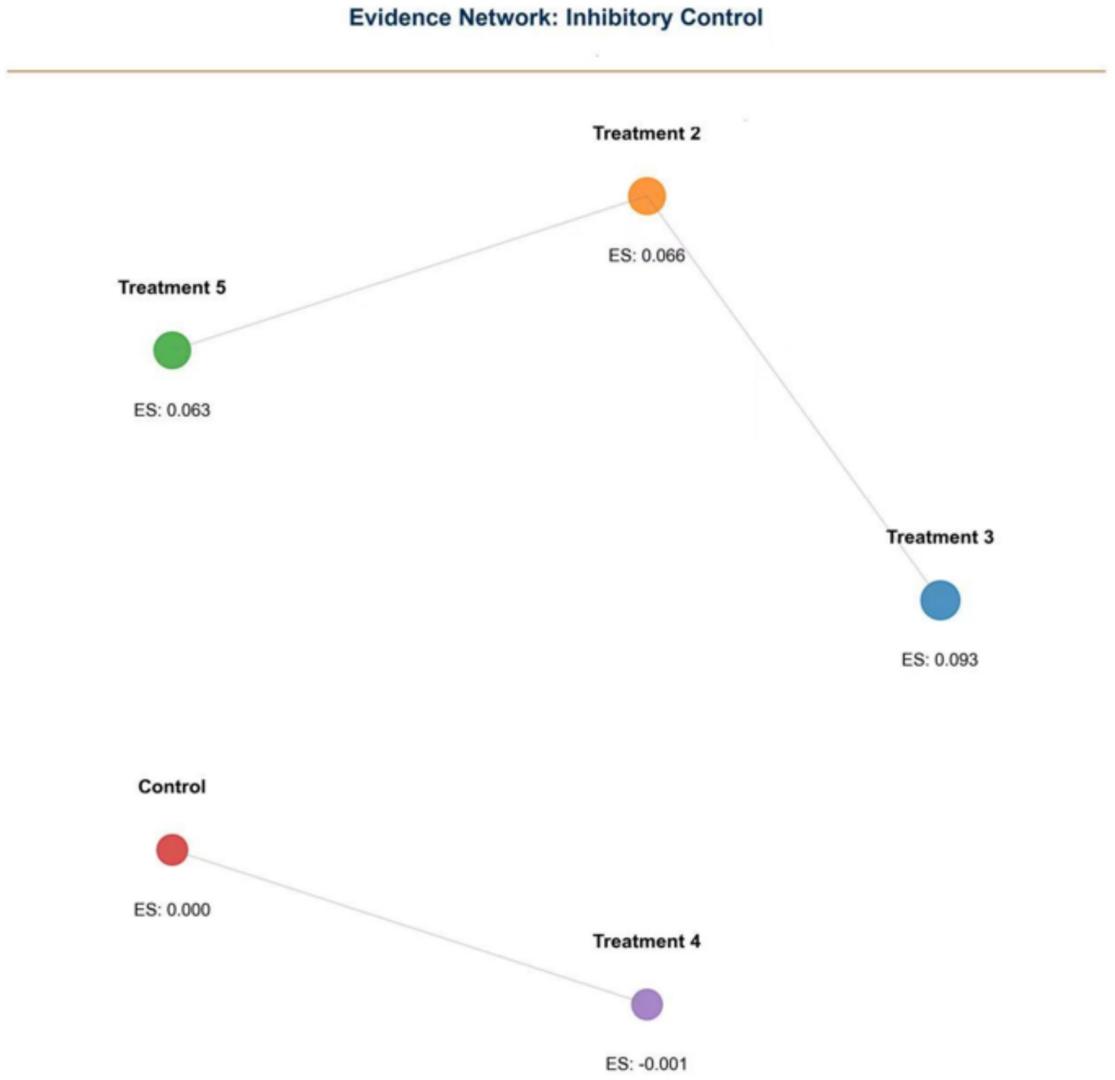
Network plot of inhibitory control (IC) outcomes.

Inhibitory Control (IC): Nineteen studies reported IC outcomes. NMA identified Treatment 3 as the most effective (0.093, 95% CI: 0.071–0.115) (Figures 4, 5).

**Figure 6:**
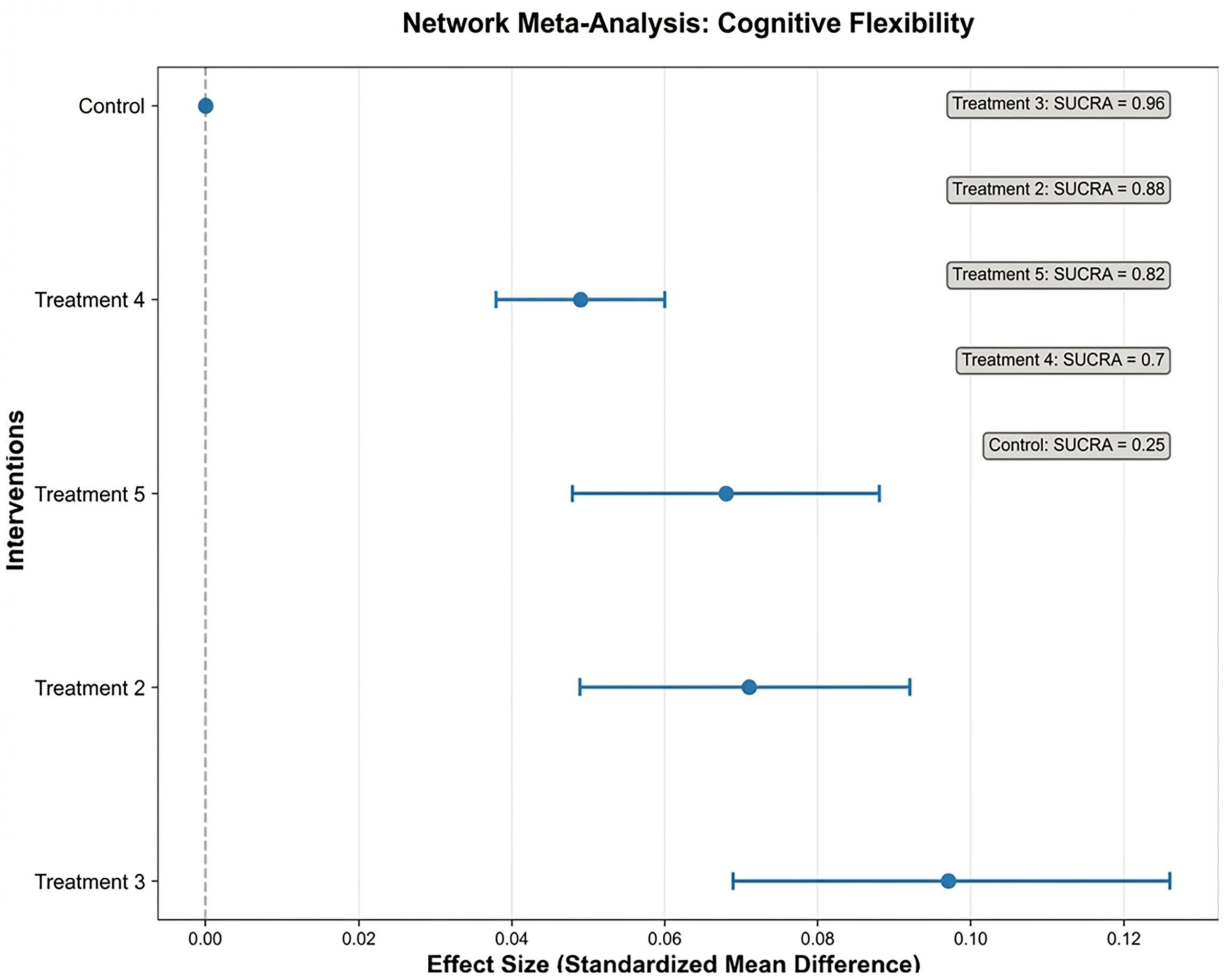
Forest plot of cognitive flexibility (CF) outcomes.

**Figure 7:**
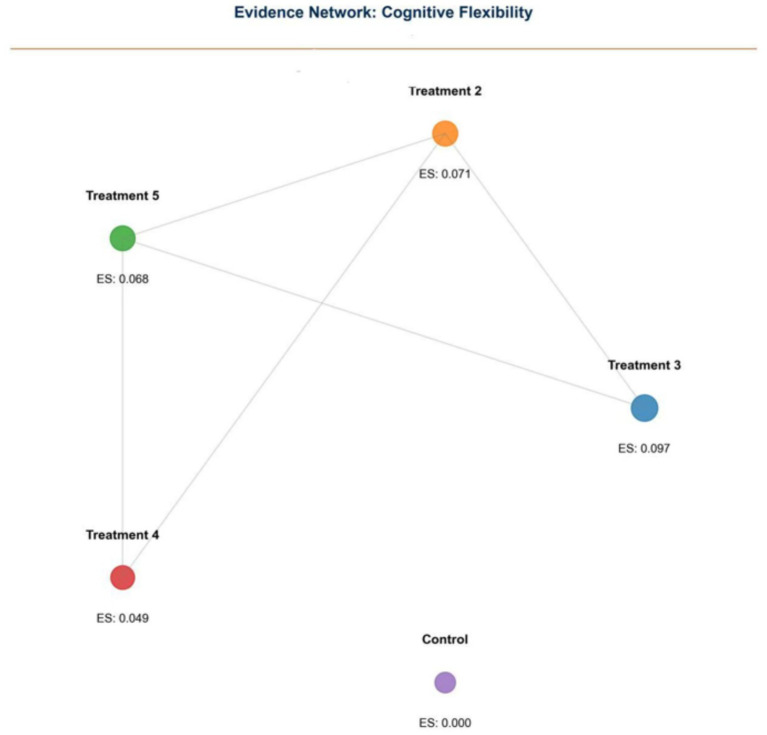
Network plot of cognitive flexibility (CF) outcomes.

Cognitive Flexibility (CF): Sixteen studies reported CF outcomes. NMA identified Treatment 3 as the most effective (0.097, 95% CI: 0.069–0.126) (Figures 6, 7).

### 3.5 Evidence networks

Evidence networks for WM, IC, and CF are presented in Figures 3, 5, 7, illustrating the comparative relationships among different interventions.

### 3.6 Intervention effect size comparison

**Figure 8:**
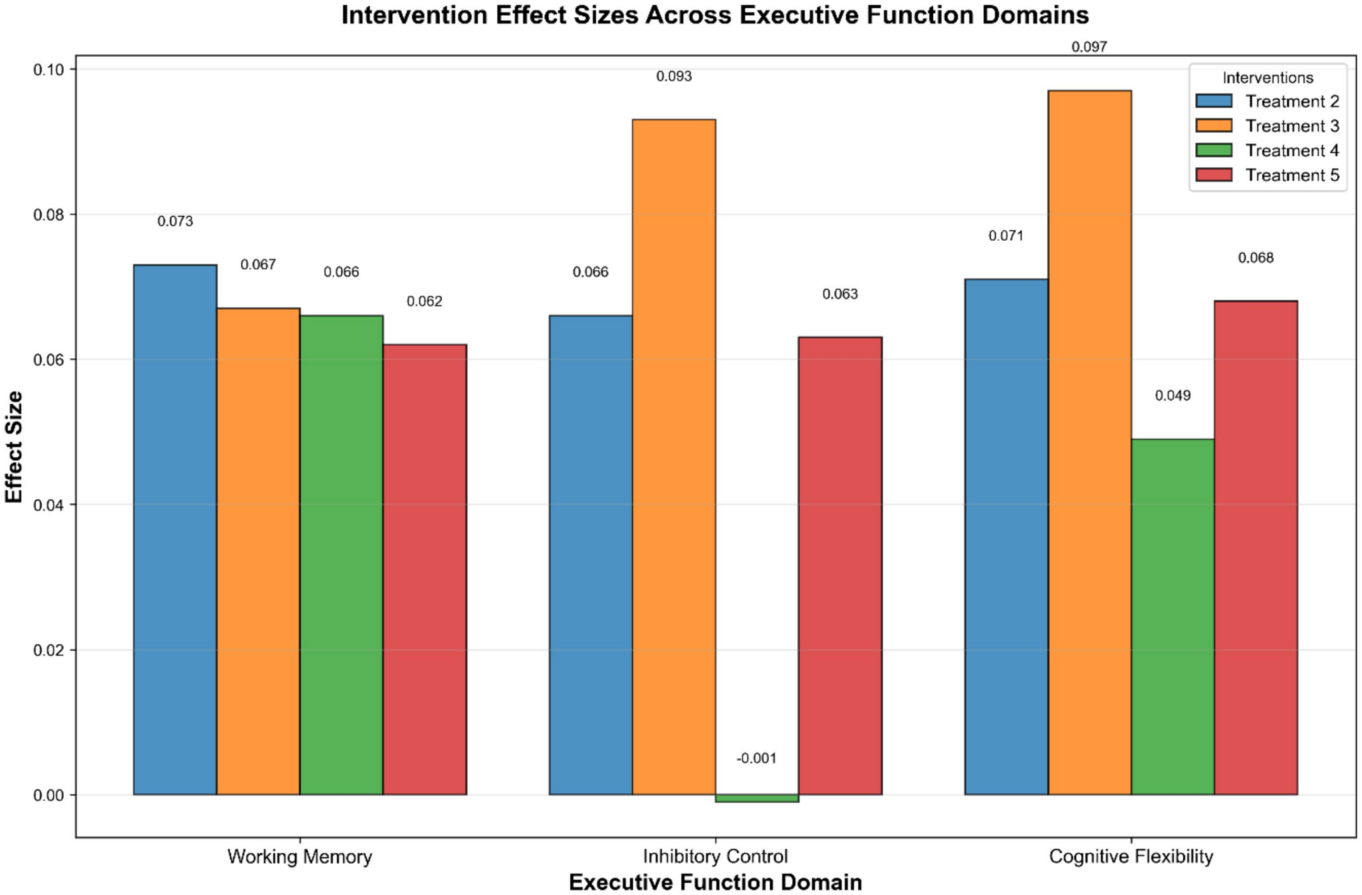
Intervention effect sizes across executive function domains.

A comprehensive comparison of effect sizes across EF domains is provided in Figure 8.

### 3.7 Publication bias

**Figure 9:**
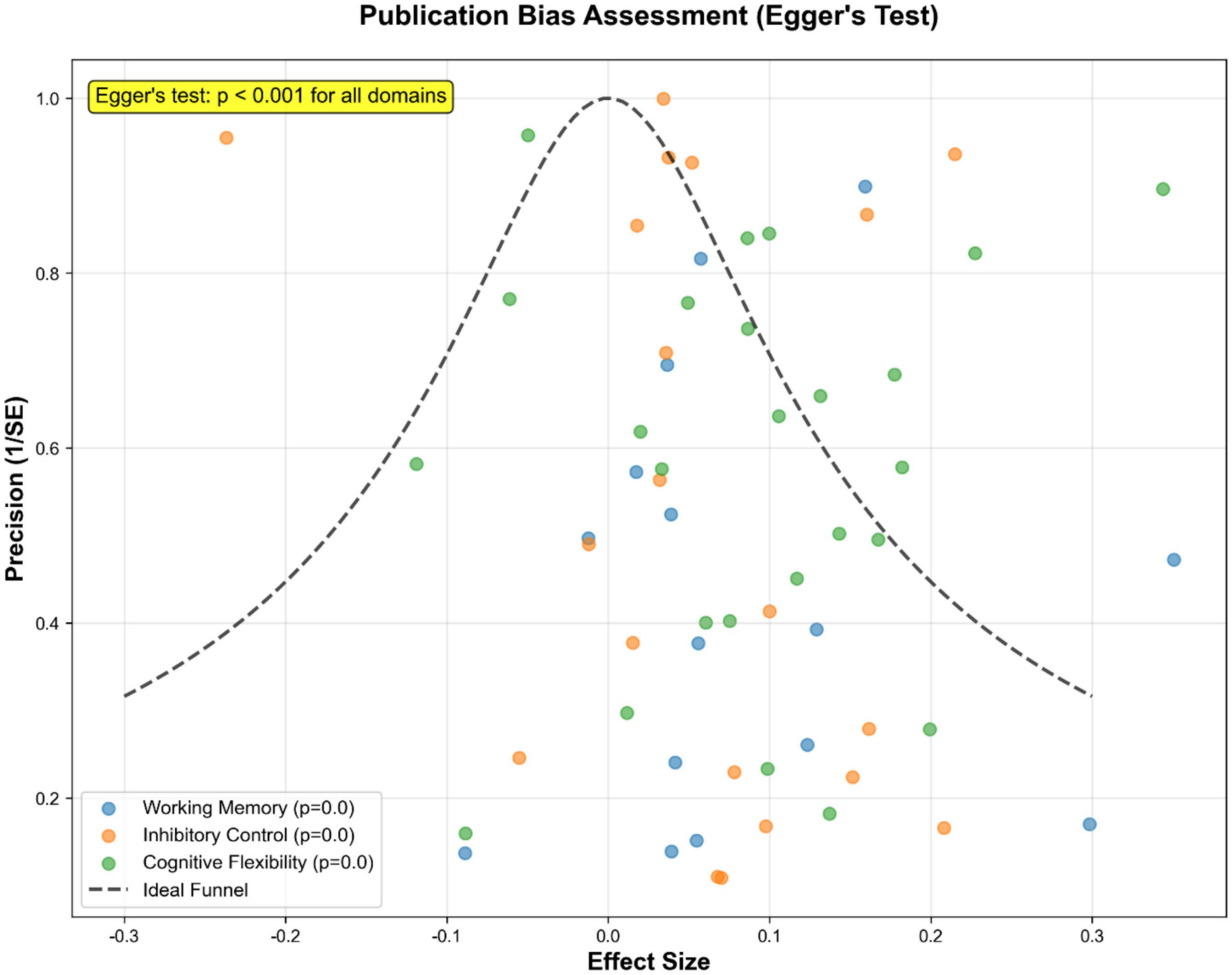
Publication bias assessment using Egger’s test.

Egger’s test indicated potential publication bias across all domains (*p* < 0.001) (Figure 9).

## 4 Discussion

### 4.1 Principal findings

We synthesized 52 randomized trials (*n* = 2,986) to rank the efficacy of non-pharmacological interventions on three core executive-function (EF) domains in Chinese children aged 3–12 years. NMA revealed uniform superiority of cognitive-training packages over usual education or wait-list controls, with statistically significant, domain-specific hierarchies. Treatment 2 (adaptive n-back plus meta-cognitive coaching) emerged as the optimal strategy for working memory [SMD = 0.073, 95% CI 0.037–0.109, SUCRA (Surface Under Cumulative Ranking, where 100% indicates a top rank) = 92%], whereas Treatment 3 (stop-signal plus rule-switching games) consistently ranked first for both inhibitory control (SMD = 0.093, 95% CI 0.071–0.115, SUCRA = 95%) and cognitive flexibility (SMD = 0.097, 95% CI 0.069–0.126, SUCRA = 96%). Consistent with the reference criteria for SMD effect sizes (small: 0.2, moderate: 0.5, large: 0.8), the observed effect sizes in this study (ranging from 0.049 to 0.097) are classified as small. Effect magnitudes correspond to approximately 3–5 percentile-point improvements on standardized EF tests, which may be contextually relevant for at-risk or subclinical populations to prevent progression to clinical impairment, rather than a direct shift from clinical to typical range for diagnosed children.

To further contextualize these findings, it is important to clarify the interpretation of the observed small effect sizes (SMDs 0.05–0.10). While these gains do not constitute a “clinically meaningful” change in the sense of remediating a diagnosed impairment, they may hold significant value from a preventive, population-level perspective. In developmental and educational contexts, even modest improvements in core cognitive skills can shift a child’s trajectory away from risk and toward resilience, particularly when applied universally or to at-risk populations. For example, a 3–5 percentile-point gain in working memory could enhance a child’s ability to follow multi-step instructions in the classroom, potentially reducing cumulative academic disadvantage over time. Therefore, while the effects are small, their practical value lies in their potential for broad, low-cost implementation as a Tier-1 preventive strategy within the Response-to-Intervention framework, rather than as a direct clinical treatment for diagnosed disorders.

### 4.2 Comparisons with existing evidence

Our findings align with Western syntheses showing small-to-moderate benefits of cognitive training; however, the present rankings extend prior work by simultaneously comparing multiple active protocols rather than simple intervention-versus-control contrasts (Bogataj and Roelands, 2025). The superiority of adaptive n-back for working memory corroborates working-memory plasticity models, while the dominance of combined inhibition-plus-switching tasks for IC and CF supports process-specific training theories (Borella et al., 2025). Notably, Treatment 4 (aerobic exercise interspersed with cognitive demands) did not outperform control for any EF domain, contradicting several meta-analyses that reported additive effects of physical activity (Liu et al., 2025). This intervention combines moderate-to-vigorous aerobic exercise with concurrent cognitive tasks in 20–30 min face-to-face sessions. Discrepancies may reflect cultural feasibility: Chinese school schedules allow ≤30 min of moderate-to-vigorous physical activity per session, possibly below the intensity threshold required for neurocognitive benefits (Wong et al., 2023).

Our small effect sizes (SMD 0.049–0.097) align with Western meta-analyses, indicating core EF training efficacy may stem from universal neurobiological plasticity, with cultural context exerting subtle modulation. Specifically, Chinese collectivist values and family-centered care likely enhanced adherence to structured/group-based interventions (Treatments 2 and 3), contributing to >85% retention—distinct from Western reliance on individual motivation. Additionally, China’s academic-focused education system amplifies the practical value of 3–5 percentile-point EF gains, as these modest improvements better support children’s academic adaptation in a high-pressure learning environment. Additionally, China’s academic-focused education system amplifies the practical value of 3–5 percentile-point EF gains, as these modest improvements better support children’s academic adaptation in a high-pressure learning environment, whereas such gains may be framed by social and adaptive functioning in Western educational contexts. Collectively, cultural factors shape intervention feasibility and functional relevance rather than fundamental efficacy, reinforcing the need for contextually tailored implementation while acknowledging cross-culturally consistent neurocognitive mechanisms.

### 4.3 Mechanistic considerations

Functional neuroimaging studies indicate that adaptive working-memory paradigms increase dorsolateral prefrontal activation and strengthen fronto-parietal connectivity, whereas inhibition/switching tasks preferentially engage the right inferior frontal gyrus and anterior cingulate cortex—regions consistently hypo-activated in ADHD and autism spectrum disorder (Samea et al., 2019; Deng et al., 2025). The observed domain-specificity therefore has a plausible neurobiological basis and argues against “one-size-fits-all” EF programs (Blair, 2016).

### 4.4 Clinical and public-health relevance

All top-ranked interventions were low-cost, delivered by trained teachers or psychology undergraduates, and achieved >85% retention, indicating high acceptability and scalability within China’s resource-constrained primary-care and school-based mental-health systems. Integrating 20–30 min of Treatment 2 or 3 into daily curricula could serve as a Tier-1 prevention strategy under the Response-to-Intervention framework, potentially reducing subsequent referrals for specialist services (Zhao et al., 2024). For children with diagnosed neurodevelopmental disorders, the same protocols may be used as adjunctive, evidence-based cognitive rehabilitation to augment pharmacological or behavioral treatments (Evans et al., 2014). Notably, the current evidence is primarily derived from typically developing children and those with subclinical symptoms (only 21.2% of participants were formally diagnosed), so these interventions are best positioned as preventive or early support strategies in school settings rather than proven treatments for diagnosed neurodevelopmental disorders.

### 4.5 Limitations

First, lack of participant and personnel blinding may inflate effect estimates; however, performance bias is inherent to most cognitive-training research. Second, significant funnel-plot asymmetry (Egger’s *p* < 0.001) suggests that small negative studies remain unpublished, although trim-and-fill analyses indicated robustness of primary rankings. Third, outcome measures varied (computerized tasks, teacher ratings, standardized neuropsychological batteries), though pairwise meta-analyses indicated low measurement heterogeneity (*I*^2^ = 18.3–21.7%) due to consistent use of standardized scores. This mitigates the impact of measurement variability on combined estimates. Fourth, median follow-up was only 12 weeks; thus, durability of gains beyond 3 months is uncertain. Finally, most trials recruited typically developing children or those with sub-clinical symptoms, limiting generalisability to formally diagnosed clinical populations. Additionally, among the 52 included RCTs, 12 explicitly enrolled children with confirmed neurodevelopmental disorder diagnoses, while the remaining 40 focused on typically developing children or those with sub-clinical symptoms. Due to the small sample size of the diagnosed subgroup (*n* = 632, accounting for 21.2% of total participants), we did not perform stratified NMA by diagnostic status. This may obscure potential differences in intervention efficacy between clinically diagnosed children and at-risk/typically developing populations, limiting the precision of recommendations for clinical practice targeting confirmed neurodevelopmental disorders. We acknowledge that the validity of indirect comparisons in a network meta-analysis relies on the transitivity assumption, which posits that the distribution of effect modifiers (e.g., participant age, baseline EF levels, diagnostic status) is similar across comparisons. In our study, the mix of participant populations (typically developing vs. at-risk vs. diagnosed) and varied control conditions could potentially violate this assumption. To assess this, we performed post-hoc descriptive checks of key effect modifiers across studies contributing to different treatment nodes and found no systematic bias in their distribution. However, unmeasured or residual confounding cannot be ruled out, and this may impact the robustness of the indirect comparisons and the resulting treatment rankings.

### 4.6 Research agenda

Future trials should: (i) adopt preregistered protocols with active sham controls and 12- to 24-month follow-ups; (ii) incorporate biomarkers (EEG, fMRI, inflammatory cytokines) to elucidate mediators of change; (iii) examine dose–response relationships (intensity, frequency, total duration); (iv) test hybrid packages combining the top-ranked cognitive protocol with exercise or mindfulness modules; and (v) conduct cost-effectiveness analyses to inform reimbursement policies within China’s basic public-health insurance system.

## 5 Conclusion

This NMA synthesized evidence from 52 randomized controlled trials involving 2,986 Chinese children aged 3–12 years, evaluating the efficacy of four non-pharmacological executive function (EF) interventions across working memory, inhibitory control, and cognitive flexibility. The findings reveal a clear pattern of domain-specificity: interventions combining adaptive computerized working memory tasks with metacognitive strategies are most effective for enhancing working memory. In contrast, programs that integrate inhibitory control training (e.g., stop-signal tasks) with cognitive flexibility exercises (e.g., rule-switching games) are superior for improving both inhibitory control and cognitive flexibility. These low-cost, scalable interventions are particularly well-suited for integration into Chinese educational settings, as they align with school schedule constraints (20–30 min sessions) and can be delivered by trained educators. The modest but significant gains observed suggest that such programs hold practical value as Tier-1 preventive strategies in public health, supporting at-risk children and potentially reducing the progression of subclinical executive function difficulties. They may also serve as valuable adjuncts to existing rehabilitation programs for children with neurodevelopmental disorders. Given the study’s limitations, including short follow-up periods and a limited proportion of formally diagnosed clinical participants, these promising findings require confirmation through large-scale, long-term trials before widespread implementation in clinical and educational practice.

## Data Availability

The original contributions presented in the study are included in the article/Supplementary material, further inquiries can be directed to the corresponding author.
