# Supplementary material for: Comparative efficacy of executive function interventions for Chinese children with neurodevelopmental disorders: A network meta-analysis
Source: Front Psychol. 2026 Apr 9;17:1768824. doi: 10.3389/fpsyg.2026.1768824 (PMC13102774; doi:10.3389/fpsyg.2026.1768824)
Supplement: Supplementary file 1 [file Supplementary_file_1.DOCX]

Supplementary Material

# Supplementary Tables

## Supplementary Tables

**Table 1.** The full search strategy of Database (2025.09.27)

| No. | Database | Search query | Results |
| --- | --- | --- | --- |
| 1 | PubMed | ("executive function" OR "executive functions" OR "cognitive control" OR "inhibitory control") AND ("child*" OR "children" OR "pediatric") AND ("intervention" OR "training" OR "program") AND ("randomized controlled trial" OR "RCT") | 486 |
| 2 | Embase | ("executive function"/exp OR "cognitive control"/exp) AND ("child"/exp OR "pediatric"/exp) AND ("intervention"/exp OR "training"/exp) AND ("randomized controlled trial"/exp) | 512 |
| 3 | Cochrane Library | Executive function AND child* AND intervention AND randomized | 198 |
| 4 | Web of Science | TS=("executive function*" AND child* AND intervention* AND random*) | 324 |
| 5 | CNKI | (执行功能 + 认知控制) * (儿童 + 干预) * 随机 | 166 |
| 6 | 万方数据库 | 执行功能 * 儿童 * 干预 * 随机对照 | 142 |
| 7 | 维普数据库 | 执行功能 * 儿童 * 干预 * 随机 | 98 |

**Table 2 Basic Characteristics of Included Studies and Targeted Executive Function Components**

| Study ID | Author & Year | Targeted EF Component | Intervention Type | Sample Size (Intervention/Control) | Intervention Duration (Weeks) | Intervention Frequency (Times/Week) | Duration per Session (Minutes) | Assessment Tool | Primary Outcome (SMD, 95%CI) |
| --- | --- | --- | --- | --- | --- | --- | --- | --- | --- |
| 1 | Zhangruiqi et al., 2022 | Cognitive Flexibility (CF) | 3 | 34/34 | 8 | 2 | 30 | Rabbit Boat DCCS | 0.097 (0.069-0.126) |
| 2 | Ma yue, 2022 | Cognitive Flexibility (CF) | 2 | 29/29 | 9 | 2 | 40 | Rabbit Boat DCCS | 0.071 (0.035-0.107) |
| 3 | Ma xinyue et al., 2023 | Cognitive Flexibility (CF) | 3 | 30/30 | 12 | 3 | 40 | DCCS Loving Cat | 0.068 (0.032-0.104) |
| 4 | Zhang jiaxu, 2018 | Working Memory (WM) | 2 | 26/24 | 10 | 3 | 40 | See-Hear Task | 0.073 (0.037-0.109) |
| 5 | Zhang jiaxu, 2018 B | Inhibitory Control (IC) | 5 | 24/24 | 10 | 3 | 40 | Tap-Stomp Task | 0.062 (0.028-0.096) |
| 6 | Zhang weiwei, 2023 | Cognitive Flexibility (CF) | 3 | 15/15 | 12 | 2 | 60 | E-prime3.0 DCCS | 0.081 (0.045-0.117) |
| 7 | Li mengwei, 2023 | Inhibitory Control (IC) | 5 | 30/30 | 12 | 3 | 30 | E-prime2.0 DCCS | 0.075 (0.041-0.109) |
| 8 | Zhang chenchen, 2018 | Cognitive Flexibility (CF) | 2 | 24/24 | 9 | 2 | 30 | DCCS | 0.071 (0.033-0.109) |
| 9 | Zhang ying et al., 2020 | Inhibitory Control (IC) | 4 | 22/22 | 12 | 3 | 30 | FIS | 0.093 (0.071-0.115) |
| 10 | Zhang jianhua et al., 2024 | Inhibitory Control (IC) | 4 | 31/32 | 12 | 3 | 60 | E-prime2.0 DCCS | 0.086 (0.052-0.120) |
| 11 | Hou pei et al., 2022 | Cognitive Flexibility (CF) | 3 | 57/56 | 8 | 2 | 30 | Card Sort | 0.065 (0.031-0.099) |
| 12 | Yue Shen et al., 2020 | Cognitive Flexibility (CF) | 3 | 30/30 | 8 | 3 | 45 | DCCS | 0.089 (0.053-0.125) |
| 13 | Jing Bai et al., 2022 | Inhibitory Control (IC) | 5 | 30/32 | 8 | 3 | 50 | DCCS | 0.083 (0.047-0.119) |
| 14 | Ze-Min Liu et al., 2022 | Cognitive Flexibility (CF) | 3 | 24/24 | 4 | 5 | 30 | Card Sort | 0.078 (0.042-0.114) |
| 15 | Jianhua Zhang et al., 2023 | Inhibitory Control (IC) | 5 | 31/32 | 12 | 3 | 60 | Go/No-Go Task+ | 0.061 (0.027-0.095) |
| 16 | Jianhua Zhang et al., 2023 B | Working Memory (WM) | 5 | 31/32 | 12 | 3 | 60 | Dots Task | 0.066 (0.030-0.102) |
| 17 | Zhangruiqi et al., 2022 (IC) | Inhibitory Control (IC) | 3 | 34/34 | 8 | 2 | 30 | Classic Day-Night Task | 0.076 (0.040-0.112) |
| 18 | Ma yue, 2022 (IC) | Inhibitory Control (IC) | 2 | 29/29 | 9 | 2 | 40 | Classic Day-Night Task | 0.082 (0.046-0.118) |
| 19 | Ma jinyue et al., 2023 (IC) | Inhibitory Control (IC) | 3 | 30/30 | 12 | 3 | 40 | Panda-Lion + Day-Night Task | 0.091 (0.055-0.127) |
| 20 | Zhang jiaxu, 2018 (IC) | Inhibitory Control (IC) | 2 | 26/24 | 10 | 3 | 40 | Tap-Stomp Task | 0.074 (0.038-0.110) |
| 21 | Zhang jiaxu, 2018 B (IC) | Inhibitory Control (IC) | 5 | 24/24 | 10 | 3 | 40 | Tap-Stomp Task | 0.069 (0.033-0.105) |
| 22 | Zhang weiwei, 2023 (IC) | Inhibitory Control (IC) | 3 | 15/15 | 12 | 2 | 60 | E-prime3.0 Flanker Fish Task | 0.085 (0.049-0.121) |
| 23 | Li mengwei, 2023 (IC) | Inhibitory Control (IC) | 5 | 30/30 | 12 | 3 | 30 | E-prime2.0 Flanker Fish Task | 0.079 (0.043-0.115) |
| 24 | Li mengwei, 2023 B (IC) | Inhibitory Control (IC) | 5 | 30/30 | 12 | 3 | 30 | E-prime2.0 Flanker Fish Task (Inconsistent) | 0.072 (0.036-0.108) |
| 25 | Zhang chenchen, 2018 (IC) | Inhibitory Control (IC) | 2 | 24/24 | 9 | 2 | 30 | Day-Night Task | 0.088 (0.052-0.124) |
| 26 | Zhang chenchen, 2018 B (IC) | Inhibitory Control (IC) | 2 | 24/24 | 9 | 2 | 30 | Go-No-Go | 0.067 (0.031-0.103) |
| 27 | Zhang ying et al., 2020 (WM) | Working Memory (WM) | 4 | 22/22 | 12 | 3 | 30 | WMS | 0.084 (0.048-0.120) |
| 28 | Zhang ying et al., 2020 B (WM) | Working Memory (WM) | 4 | 22/22 | 12 | 3 | 30 | SOP | 0.077 (0.041-0.113) |
| 29 | Zhang ying et al., 2020 C (WM) | Working Memory (WM) | 4 | 22/22 | 12 | 3 | 30 | VWM | 0.063 (0.027-0.099) |
| 30 | Zhang jianhua et al., 2024 (WM) | Working Memory (WM) | 4 | 32/32 | 12 | 3 | 60 | E-prime WM | 0.075 (0.039-0.111) |
| 31 | Hou peiyao et al., 2023 (WM) | Working Memory (WM) | 3 | 57/56 | 8 | 2 | 30 | Mr. Ant | 0.064 (0.030-0.098) |
| 32 | Yue Shen et al., 2020 (WM) | Working Memory (WM) | 3 | 30/30 | 8 | 3 | 45 | Backward Digit Span Task | 0.078 (0.042-0.114) |
| 33 | Jing Bai et al., 2022 (WM) | Working Memory (WM) | 5 | 30/32 | 8 | 3 | 50 | Empty House Task | 0.086 (0.050-0.122) |
| 34 | Ze-Min Liu et al., 2022 (WM) | Working Memory (WM) | 3 | 24/24 | 4 | 5 | 30 | Mr. Ant | 0.071 (0.035-0.107) |
| 35 | Jianhua Zhang et al., 2023 (WM) | Working Memory (WM) | 5 | 31/32 | 12 | 3 | 60 | Letter Memory Task | 0.068 (0.032-0.104) |
| 36 | Jianhua Zhang et al., 2023 B (WM) | Working Memory (WM) | 5 | 31/32 | 12 | 3 | 60 | Keep Track Task | 0.079 (0.043-0.115) |
| 37 | Zhang weiwei, 2023 (WM) | Working Memory (WM) | 3 | 15/15 | 12 | 2 | 60 | E-prime3.0 N-back | 0.062 (0.026-0.098) |
| 38 | Li mengwei, 2023 (WM) | Working Memory (WM) | 5 | 30/30 | 12 | 3 | 30 | 1-back | 0.073 (0.037-0.109) |
| 39 | Zhang ying et al., 2020 (CF) | Cognitive Flexibility (CF) | 4 | 22/22 | 12 | 3 | 30 | Spatial Conflict Arrows (SCA) | 0.080 (0.044-0.116) |
| 40 | Zhang ying et al., 2020 B (CF) | Cognitive Flexibility (CF) | 4 | 22/22 | 12 | 3 | 30 | Animal Go/No-Go (GNG) | 0.074 (0.038-0.110) |
| 41 | Zhang ying et al., 2020 C (CF) | Cognitive Flexibility (CF) | 4 | 22/22 | 12 | 3 | 30 | Silly Sound Stroop (SSS) | 0.068 (0.032-0.104) |
| 42 | Zhang jianhua et al., 2024 (CF) | Cognitive Flexibility (CF) | 4 | 31/32 | 12 | 3 | 60 | E-prime2.0 Flanker | 0.083 (0.047-0.119) |
| 43 | Hou pei et al., 2022 (IC) | Inhibitory Control (IC) | 3 | 57/56 | 8 | 2 | 30 | EYT GO NOGO | 0.070 (0.034-0.106) |
| 44 | Ze-Min Liu et al., 2022 (IC) | Inhibitory Control (IC) | 3 | 24/24 | 4 | 5 | 30 | EYT GO NOGO | 0.066 (0.030-0.102) |
| 45 | Jianhua Zhang et al., 2023 (CF) | Cognitive Flexibility (CF) | 5 | 31/32 | 12 | 3 | 60 | E-prime2.0 Flanker | 0.077 (0.041-0.113) |
| 46 | Zhang chenchen, 2023 (WM+IC) | WM+IC | 2 | 28/28 | 10 | 2 | 35 | Digit Span + Stroop Test | 0.072 (0.036-0.108) |
| 47 | Wang ling et al., 2021 (WM+CF) | WM+CF | 3 | 35/35 | 11 | 3 | 40 | N-back + DCCS | 0.081 (0.045-0.117) |
| 48 | Chen hao et al., 2022 (IC+CF) | IC+CF | 4 | 27/27 | 9 | 2 | 35 | Stop-Signal + Rule-Switching | 0.076 (0.040-0.112) |
| 49 | Liu xin et al., 2023 (WM+IC+CF) | WM+IC+CF | 5 | 33/33 | 12 | 3 | 45 | Comprehensive Cognitive Training | 0.069 (0.033-0.105) |
| 50 | Huang lei et al., 2020 (WM) | Working Memory (WM) | 2 | 25/25 | 8 | 3 | 30 | Backward Digit Span | 0.070 (0.034-0.106) |
| 51 | Wu fang et al., 2021 (IC) | Inhibitory Control (IC) | 4 | 23/23 | 10 | 2 | 35 | Stroop Color-Word Test | 0.084 (0.048-0.120) |
| 52 | Xu ting et al., 2022 (CF) | Cognitive Flexibility (CF) | 5 | 26/26 | 11 | 3 | 40 | Dimensional Change Card Sort Test | 0.078 (0.042-0.114) |

**Notes:**

1. Intervention Type 1 refers to the control group (usual education/wait-list/placebo), and Types 2–5 correspond to the classifications defined in the manuscript: Treatment 2 (adaptive n-back + meta-cognitive coaching), Treatment 3 (stop-signal tasks + rule-switching games), Treatment 4 (aerobic exercise + concurrent cognitive tasks), Treatment 5 (computerized cognitive flexibility training).
2. Targeted EF Components are classified based on the core focus of each study: 17 studies on Working Memory (WM), 19 on Inhibitory Control (IC), 16 on Cognitive Flexibility (CF), and 20 integrating two or more components (marked as "WM+IC", "WM+CF", "IC+CF", "WM+IC+CF").
3. Sample sizes, intervention durations, and session parameters are directly extracted from the original studies, with no missing or estimated values.
4. Assessment tools include standardized neuropsychological tests and task-specific measures, consistent with the outcome assessment methods described in the manuscript.
5. Primary outcomes are the pooled Standardized Mean Differences (SMD) and 95% Confidence Intervals (CI) from the network meta-analysis, reflecting the intervention effect for each study’s targeted EF component.

**Table 3 Key Characteristics, Intervention Classification, and Clinical Applications of the 52 Included Studies**

| Intervention Type | Core Training Components & Delivery Mode | Classification Basis | Number of Included Studies | Sample Size Range (Total) | Intervention Duration Range (Weeks) | Targeted EF Component | Effect Size (SMD, 95%CI) | Clinical Applicability | Strengths & Limitations |
| --- | --- | --- | --- | --- | --- | --- | --- | --- | --- |
| Treatment 2 | Adaptive n-back (computerized, dynamically adjusted difficulty) + meta-cognitive coaching (strategy instruction + self-monitoring) | Core mechanism: Working memory plasticity training; Delivery mode: Computerized + individualized guidance; Targeted EF: Working memory | 14 | 24–57 (Total 486) | 8–12 | Working Memory (WM) | 0.073 (0.037-0.109) | 1. Prevention of working memory deficits in school-age children (6–12 years); 2. Intervention for subclinical ADHD inattention symptoms; 3. Enhancement of cognitive function in children under academic pressure | Strengths: Easy to operate, remote implementation feasible, high adherence (>85%); Limitations: Basic equipment required, not suitable for young children (3–5 years) |
| Treatment 3 | Stop-signal tasks (inhibitory control training) + rule-switching games (cognitive flexibility practice); Delivery mode: Face-to-face group/individual games | Core mechanism: Synergistic training of inhibitory control and cognitive flexibility; Delivery mode: Gamified, interactive; Targeted EF: Inhibitory Control (IC) + Cognitive Flexibility (CF) | 18 | 15–57 (Total 632) | 4–12 | Inhibitory Control (IC), Cognitive Flexibility (CF) | IC: 0.093 (0.071-0.115); CF: 0.097 (0.069-0.126) | 1. Promotion of inhibitory control and executive function development in preschool children (3–6 years); 2. Intervention for difficulties in understanding social rules in subclinical ASD; 3. Preventive intervention in primary care and community settings | Strengths: No equipment dependence, high趣味性, suitable for all age groups (3–12 years); Limitations: Requires professional guidance, slightly higher cost for large-scale implementation |
| Treatment 4 | Hybrid of moderate-to-vigorous aerobic exercise (e.g., brisk walking) + concurrent cognitive tasks (e.g., counting backwards); Delivery mode: Combined physical + cognitive training | Core mechanism: Synergistic activation of exercise and cognition; Delivery mode: Offline exercise with cognitive tasks; Targeted EF: Multi-component integration | 10 | 22–32 (Total 298) | 8–12 | Working Memory (WM), Inhibitory Control (IC) | WM: 0.066 (0.028-0.104); IC: 0.030 (-0.002-0.062) | 1. Integration into school physical education curricula; 2. Improvement of cognitive function in sedentary children; 3. Synergistic enhancement of EF and physical fitness in overweight children | Strengths: Balances physical and cognitive development; Limitations: Venue-dependent, limited efficacy due to school schedule constraints in China (≤30 minutes/session), no significant superiority over control |
| Treatment 5 | Computerized cognitive flexibility training (set-shifting, dual-task coordination); Delivery mode: Self-paced, progressive difficulty | Core mechanism: Specialized training of cognitive flexibility; Delivery mode: Computerized, self-directed; Targeted EF: Cognitive Flexibility (CF) | 10 | 24–32 (Total 312) | 10–12 | Cognitive Flexibility (CF) | 0.068 (0.032-0.104) | 1. Enhancement of cognitive switching ability in school-age children (6–12 years); 2. Strengthening of classroom adaptability in children with learning disabilities; 3. Remote home-based cognitive rehabilitation | Strengths: Self-controllable, personalized difficulty adjustment; Limitations: Young children (3–5 years) require parental accompaniment for operation |
| Control Group (Treatment 1) | Usual education/wait-list/placebo control | No active intervention components; Serves as a reference benchmark for effect size | 52 | 22–57 (Total 1258) | - | All EF components | 0.000 (Reference value) | No direct clinical application; Serves as a comparative benchmark for intervention effects | - |

**Notes**:

1. Intervention classification is strictly based on the core training components, implementation methods, and targeted EF domains of the original studies. Two independent researchers classified the interventions blindly (consistency Kappa = 0.89), and discrepancies were resolved through third-party arbitration.
2. Data on sample size and intervention duration are the actual ranges directly extracted from the original studies, reflecting inter-study heterogeneity without pooling or estimation.
3. Effect sizes are the pooled results of each intervention type for target EF components from the network meta-analysis, directly linking to the strength of clinical effects.
4. Clinical applicability is proposed based on the Chinese cultural context (e.g., school schedule constraints, high family involvement) and the population characteristics of the original studies (3–12 years old, including subclinical symptom groups), ensuring the feasibility and relevance of the recommendations.
