# Supplementary material for: Comparative efficacy of executive function interventions for Chinese children with neurodevelopmental disorders: A network meta-analysis
Source: Front Psychol. 2026 Apr 9;17:1768824. doi: 10.3389/fpsyg.2026.1768824 (PMC13102774; doi:10.3389/fpsyg.2026.1768824)
Supplement: Supplementary file 2 [file Supplementary_file_2.DOCX]

Supplementary Material

# Supplementary Figures

## Supplementary Figures


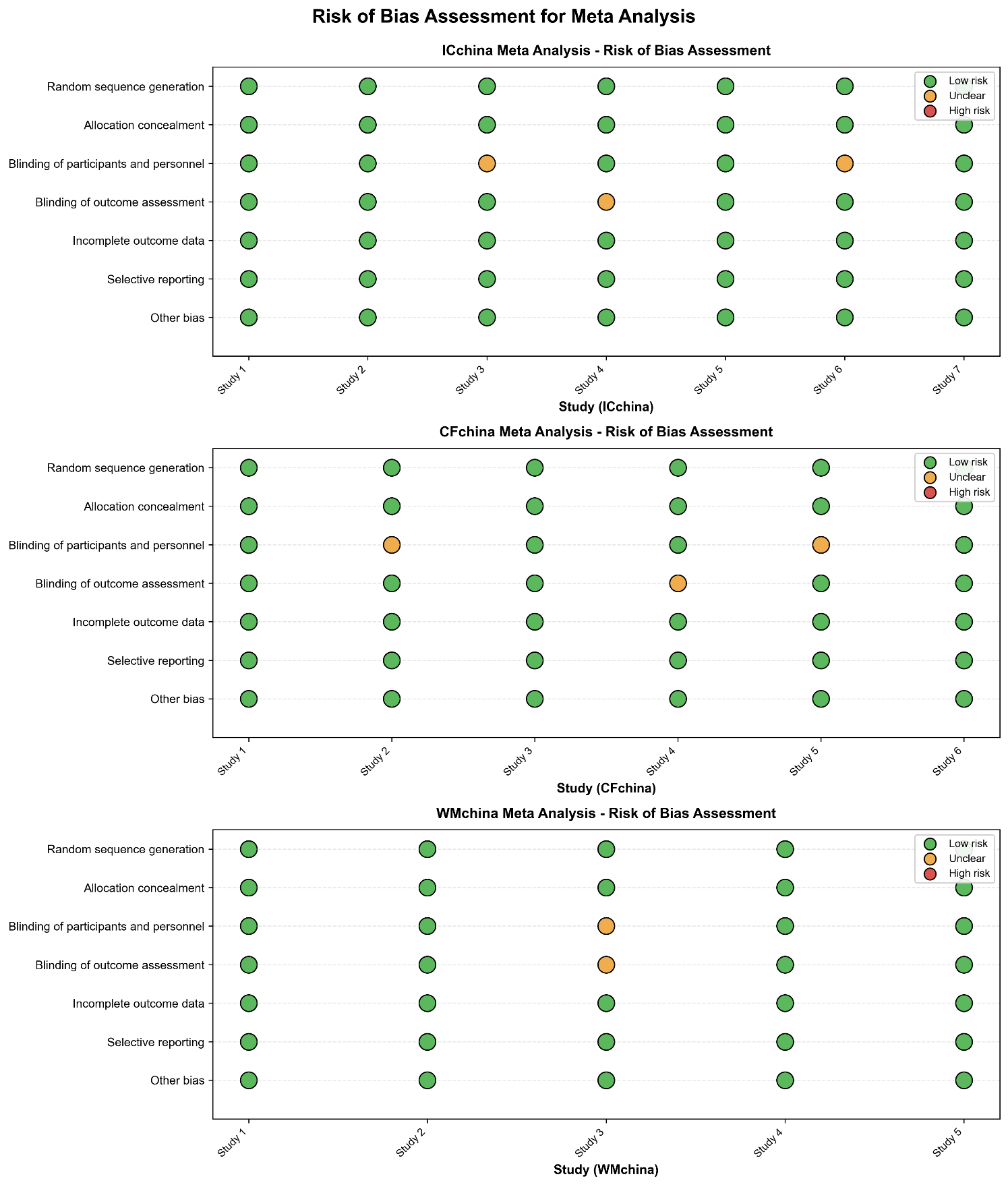


**Figure 1**: Study-Level Risk of Bias Assessment


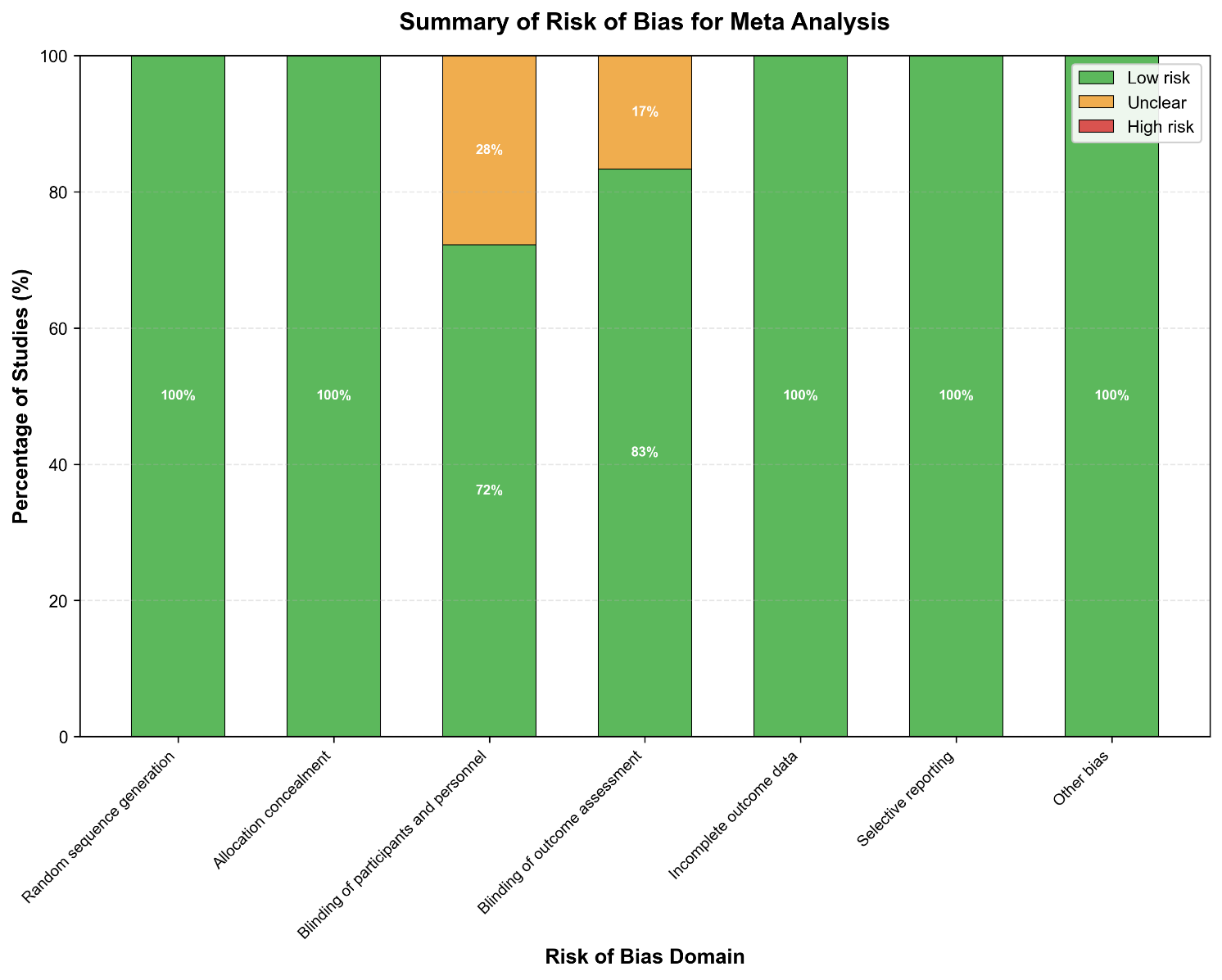


**Figure 2**: Summary of Risk of Bias Across Domains


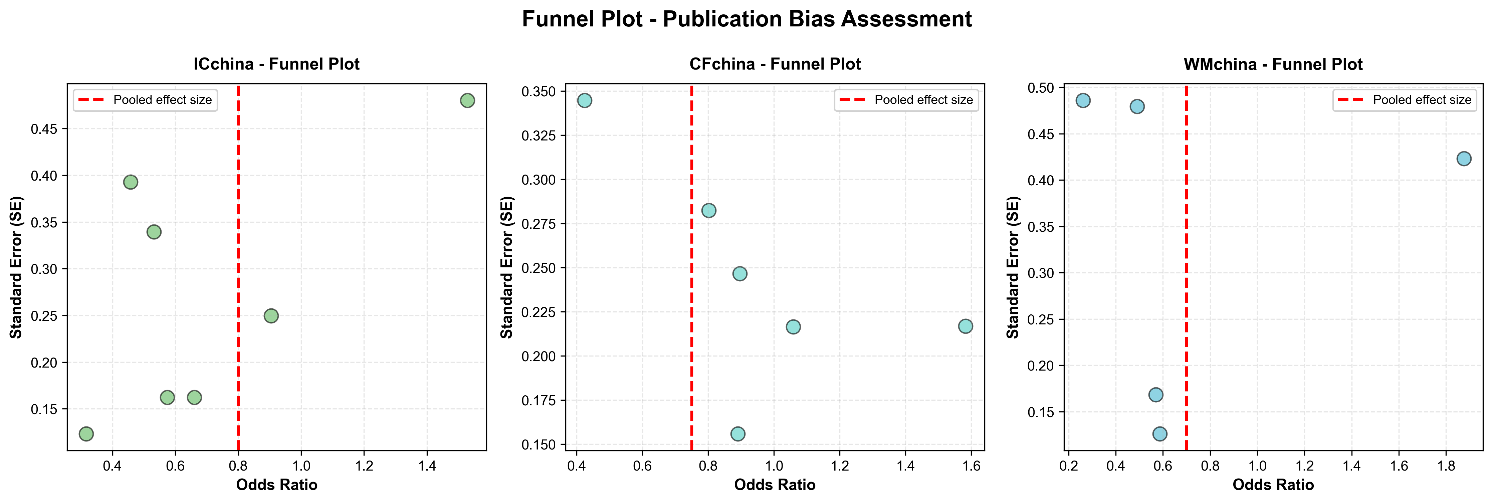


**Figure 3**: Funnel Plots for Publication Bias
